# Longitudinal Screening for Diabetic Retinopathy in a Nationwide Screening Program: Comparing Deep Learning and Human Graders

**DOI:** 10.1155/2020/8839376

**Published:** 2020-12-15

**Authors:** Jirawut Limwattanayingyong, Variya Nganthavee, Kasem Seresirikachorn, Tassapol Singalavanija, Ngamphol Soonthornworasiri, Varis Ruamviboonsuk, Chetan Rao, Rajiv Raman, Andrzej Grzybowski, Mike Schaekermann, Lily H. Peng, Dale R. Webster, Christopher Semturs, Jonathan Krause, Rory Sayres, Fred Hersch, Richa Tiwari, Yun Liu, Paisan Ruamviboonsuk

**Affiliations:** ^1^Department of Ophthalmology, College of Medicine, Rangsit University, Rajavithi Hospital, Bangkok, Thailand; ^2^Department of Ophthalmology, Chulabhorn Hospital, HRH Princess Chulabhorn College of Medical Science, Chulabhorn Royal Academy, Bangkok, Thailand; ^3^Department of Tropical Hygiene, Faculty of Tropical Medicine, Mahidol University, Bangkok, Thailand; ^4^Department of Biochemistry, Faculty of Medicine, Chulalongkorn University, Bangkok, Thailand; ^5^Shri Bhagwan Mahavir Vitreoretinal Services, Sankara Nethralaya, Chennai, Tamil Nadu, India; ^6^Department of Ophthalmology, University of Warmia and Mazury, Olsztyn, Poland; ^7^Institute for Research in Ophthalmology, Foundation for Ophthalmology Development, Poznan, Poland; ^8^Google Health, Palo Alto, CA, USA; ^9^Work done at Google via Optimum Solutions Pte Ltd, Singapore

## Abstract

**Objective:**

To evaluate diabetic retinopathy (DR) screening via deep learning (DL) and trained human graders (HG) in a longitudinal cohort, as case spectrum shifts based on treatment referral and new-onset DR.

**Methods:**

We randomly selected patients with diabetes screened twice, two years apart within a nationwide screening program. The reference standard was established via adjudication by retina specialists. Each patient's color fundus photographs were graded, and a patient was considered as having sight-threatening DR (STDR) if the worse eye had severe nonproliferative DR, proliferative DR, or diabetic macular edema. We compared DR screening via two modalities: DL and HG. For each modality, we simulated treatment referral by excluding patients with detected STDR from the second screening using that modality.

**Results:**

There were 5,738 patients (12.3% STDR) in the first screening. DL and HG captured different numbers of STDR cases, and after simulated referral and excluding ungradable cases, 4,148 and 4,263 patients remained in the second screening, respectively. The STDR prevalence at the second screening was 5.1% and 6.8% for DL- and HG-based screening, respectively. Along with the prevalence decrease, the sensitivity for both modalities decreased from the first to the second screening (DL: from 95% to 90%, *p* = 0.008; HG: from 74% to 57%, *p* < 0.001). At both the first and second screenings, the rate of false negatives for the DL was a fifth that of HG (0.5-0.6% vs. 2.9-3.2%).

**Conclusion:**

On 2-year longitudinal follow-up of a DR screening cohort, STDR prevalence decreased for both DL- and HG-based screening. Follow-up screenings in longitudinal DR screening can be more difficult and induce lower sensitivity for both DL and HG, though the false negative rate was substantially lower for DL. Our data may be useful for health-economics analyses of longitudinal screening settings.

## 1. Introduction

Blindness from diabetes is expected to rise dramatically in this new decade [1]. To reduce diabetes-associated blindness, nationwide systematic screening for diabetic retinopathy (DR) has been implemented [2]. Many countries have studied the development of systematic screening programs [3–6], resulting in several lessons learnt. First, though a large proportion of patients with well-controlled diabetes showed no retinopathy with low risk of visual loss over the years [7], nonattendance in screening programs increased risk of visual loss from sight-threatening DR (STDR) [8]. While annual DR screening is generally recommended [9, 10], studies in some resource-rich countries have found a ceiling uptake of patients [11] which was compromised by an abundance of resource investment [12]. Extending the screening interval from annual to once every 2-3 years was found to be cost-effective in several studies in Europe [13, 14].

Automated retinal disease assessment tools have been studied for DR screening since before the commercial availability of digital retinal photography [15]. Using conventional methods of machine learning, this tool reached a plateau for detecting referable DR with high sensitivity (90%) but less-stellar specificity (45%) [16] in the early 2010s. Deep learning (DL), a subfield of machine learning, has recently demonstrated robust performance with very high sensitivity (95%) and specificity (95%) [17]. Most cross-sectional studies on DL for DR screening have demonstrated this level of performance [17–21]. As a result, DR screening trends have shifted towards the use of DL in assisting or replacing trained human graders (HG) for detecting referrals in DR screening programs [18].

To assess the roles of DL in longitudinal screening for DR, a study on longitudinal performance of DL is important, particularly if the screening was to be repeated in subsequent visits. The continual screening for DR in subsequent years would encounter a shift in the case spectrum since patients correctly detected to have referable DR or STDR would be referred for treatment and exit the screening program. The cohort of patients rescreened in the following years should contain mainly cases that did not display findings of STDR in the previous screenings but might have developed new subtle changes of early STDR in the following screenings. These subtle changes may be more difficult to detect than the more obvious findings associated with well-established STDR.

In this study, we used a real-world, nationwide, longitudinal screening program for DR as a model to assess biennial screening for DR using DL and HG to grade color retinal photographs. The objective was to analyze possible changes in various screening outcomes for detecting STDR determined by DL over two years and compare them with those determined by HG.

## 2. Methods

This study utilized demographic information, laboratory data, and retinal fundus photographs from patients with diabetes in 13 health regions in the Thai national DR screening program. All data were deidentified. This study was conducted according to the Declaration of Helsinki with approvals from the Institutional Review Board of hospitals where the patients were recruited.

Instituted in 2013 by the Ministry of Public Health, the Thai DR screening program has been implemented in every province and conducted by the Noncommunicable Disease Unit in each Provincial Health Office. All patients with diabetes can access this program without cost thanks to the Universal Coverage insurance scheme provided by the National Health Security Office. Consistent with level 1 evidence suggesting its adequacy, this program employs nonmydriatic, single-field (45-degree, macular-centered) color fundus photography [22] as a screening tool with gradings by trained HG in each region to determine referral to ophthalmologists.

Our study included randomly selected patients in the DR screening program who underwent DR screening twice, two years apart (years 2014 and 2016 or 2015 and 2017). All patients had color retinal photographs of the both eyes taken at each screening. The color retinal photographs were captured by various fundus cameras: Topcon TRC-NW8, Nidek (AFC-210 and AFC-230), and KOWA (Nonmyd *α*-DIII 8300, Nonmyd 7, VX-10*α*, Nonmyd *α*-DIII, Nonmyd WX, VX-20). The diagnosis of DR was based on grading of the retinal photographs. Each photograph was graded for its DR severity level and the presence or absence of diabetic macular edema (DME) according to the International Clinical Classification of DR. The reference standard grades were provided via adjudication by three international retina specialists (from USA, India, and Thailand). As part of the study, we compared gradings from a DL system and HG to this reference standard. The HG were selected from regional DR graders within the national DR screening program. Details of gradings by the retinal specialists, DL, and HG were described previously [19].

Patients were excluded from this study if they had retinal diseases other than DR which precluded diagnosis of DR in either eye, did not have gradings from all three modalities, or if the reference standard, DL, or HG found the images ungradable. Patients were labelled as ungradable if the both eyes were ungradable, or if either eye was ungradable or the fellow eye did not have severe non-proliferative DR (NPDR), proliferative DR (PDR), or DME.

In this study, we studied a simulated setting where each patient was assigned a DR severity level based on the severity of the worse eye. Patients were labelled as STDR if either eye had either DME, severe NPDR or PDR. Those with STDR in the first screening were “referred out” for treatment and excluded from the second screening.

### 2.1. Statistical Analysis

We estimated the sample size for the first screening of no less than 5,530 patients, considering a margin of error of 10%, type 1 error at 0.05 and type 2 error at 0.2, and an STDR prevalence in Thailand of approximately 6.5% of all patients with diabetes screened for DR [23]. The number of patients included from each of the 13 health regions in the sample was proportional to the number of patients with diabetes in each region [19].

We then computed the prevalence, incidence rate, sensitivity, specificity, positive predictive value, negative predictive value, and accuracy, as well as the number and proportion of true positives, false positives, true negatives, and false negatives. The chi-squared test was used to evaluate statistical significance, with *α* = 0.05.

## 3. Results

**Figure 1:**
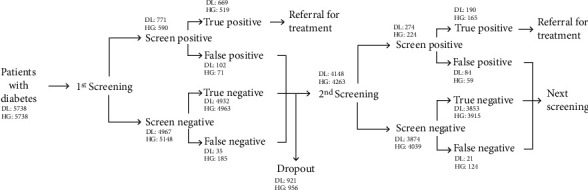
Flow of patients from the first to the second screening. The number of patients in the cohorts of deep learning (DL) and trained human graders (HG) is compared at each point of the screening. The reference standard for these cases was based on an overread by retina specialists (Methods). Screen positive/negative indicates patients whom the DL or HG indicated as positive/negative. In this simulated setting, only patients who were confirmed by retina specialists to have STDR (i.e., true positives) were referred for treatment. The remaining patients were entered into the second screening. Dropout before the second screening included patients with missing data in either DL or HG or determined as ungradable by the reference standard during the second screening.

In this retrospective study, we examined 5,738 patients who were screened for DR on two separate occasions, approximately two years apart and simulated scenarios where either the DL or HG screened for STDR. To mimic a realistic scenario, all cases who were indicated for referral by either DL or HG were verified by retina specialists (our reference standard), and only patients with verified STDR were “referred” out of the screening program (Figure 1, additional details below). Patient demographics, including prevalence of DR of different severities and DME at each screening, are shown in Table 1.

### 3.1. Comparison between DL and HG at the First Screening

At the first screening, prevalence of STDR in both the DL and HG cohorts was 12.3% (704 out of 5,738; the cohorts have yet to diverge based on the screening outcome). The DL arm indicated a greater number of cases than HG as positive for STDR (771 vs. 590, corresponding to 13% and 10% of the cohort), resulting in a substantially higher sensitivity (95% vs. 74%). Specificities of both arms was high at 98-99%. Detailed results for positive predictive value, negative predictive value, and accuracy are presented in Table 2, and the full 2 × 2 contingency table (also termed “confusion matrix”) is presented in Table 3.

### 3.2. Cohort Changes at the Second Screening

After the first screening, cases indicated as positive by the DL or HG were reviewed by retina specialists, and cases confirmed to have STDR were “referred out.” This resulted in different numbers of patients and a different case spectrum presenting for the second screening in the DL and HG arms of the study: 4,148 and 4,263 (72% and 74% of the original 5,738 patients), respectively.

During the intervening period between screenings, 195 patients developed new STDR according to the reference standard, with the majority of these cases arising from patients with moderate NPDR during the first screening (Table 4). Looking across the whole cohort, the rates of STDR were substantially higher with increasing severity of DR at the first screening: 2% for no DR, 9% for mild NPDR, and 25% for moderate NPDR. This trend of increasing 2-year STDR incidence with DR severity was also preserved when stratifying patients based on the DL and HG grades at the first screening.

Despite the approximately 200 new STDR cases, because many true positive STDR cases were referred out (669 for DL and 519 for HG), the prevalence of STDR was substantially lower in the second screening than the first screening (DL arm: 5.1% vs. 12%, *p* < 0.001; HG arm: 6.8% vs. 12.3%, *p* < 0.001).

### 3.3. Comparison between First and Second Screening for DL and HG

Consistent with the prevalence changes, the rates of positive screens by the DL and HG were both significantly lower in the second screen than in the first (DL: 6.6% vs. 13%, *p* < 0.001; HG: 5.3% vs. 10%, *p* < 0.001). The sensitivity of the DL and HG was also both lower than at their first screening, at 90% (vs. 95%, *p* = 0.008) and 57% (vs. 74%, *p* < 0.001), respectively. For both DL and HG, the specificity remained high at 98-99% without significant changes (*p* = 0.742). The positive predictive value decreased in both arms (DL: from 87% to 69%, *p* < 0.001; HG: from 88 to 74%, *p* < 0.001). Negative predictive value remained at 99% for DL and 96-97% for HG, and accuracy remained at 97-98% for DL and 96% for HG; neither of these trends were statistically significant at the *ɑ* = 0.05 level. Confidence intervals are presented in Table 2.

When examining the full contingency table (Table 3), the fraction of true positives and true negatives differed significantly between the first and second screenings; the fraction of false positives and false negatives was not statistically significantly different. This trend was consistent in both the DL and HG arms.

### 3.4. Breakdown of STDR into DR and DME

Next, we examined the prevalence of severe NPDR and PDR vs. DME among the STDR cases and among the false negatives (Supplementary Table 1). Of all STDR cases, over 91% were due to DME in the first screening as well as in both arms of the second screening. When examining the false negatives specifically, rates of DME were around 90% for HG. For DL, there were only 35 and 11 false negatives in the first and second screening, respectively; the rates of DME in the two screenings were 94% and 64%, respectively.

A similar breakdown for the non-STDR cases is presented in Supplementary Table 2, showing that among all non-STDR cases, fewer than 7% were moderate NPDR without DME. For the false positive cases specifically, a much greater proportion were moderate NPDR without DME: 65% and 54% for DL and 18% and 20% for HG.

### 3.5. Performances of DL and HG at the Eye Level

Finally, we explored the STDR detection performance of DL and HG at the eye level (Supplementary Table 3). Similar trends were observed for both DL and HG: sensitivity and positive predictive value for STDR decreased on the second screening compared to the first screening, while specificity, negative predictive value, and accuracy remained similar. The trends for considering DME and severe NPDR/PDR separately were similar.

## 4. Discussion

Globally, it is estimated that Asia-Pacific accounts for the majority of patients with poor DR-induced visual outcomes, including both blindness (51%, *n* = 424,400) and visual impairment (56%, *n* = 2.1 million) [24]. To improve DR-related visual outcomes, several countries have established DR screening programs. In our study, we conducted a longitudinal analysis of data from the Thai national DR screening program.

Our DR screening program's endpoint of interest is “STDR” (severe NPDR, PDR or DME [25]). However, we note that other definitions exist (e.g., moderate NPDR or worse [7]), which can hinder comparisons across studies. In our study, the prevalence of STDR during the first screening was 12.3%, which is comparable to the prevalence of STDR estimated from a meta-analysis of 35 studies (10.2%) [26]. As part of a longitudinal analysis, we observed 2-year incident STDR rates of 1.7% and 8.6% among patients without DR and mild DR, respectively, and 3.9% across all non-STDR patients. For comparison, a meta-analysis of 17 studies found that patients without DR and mild DR at baseline had average STDR incidence rates of approximately 1% and 8% per year, respectively [27]. Trends were similar in another study in Asia, where the incidence rate was 1.5% per year in patients without retinopathy at baseline and 13.6% at 4 years [28]. Others have reported a 4-year incidence of 1.45% from no DR at baseline and a rate of 5.02% from all cases (with or without DR) [29].

Given prior work showing that DL can be used to help detect STDR, our study focused on better understanding the longitudinal implications of using DL, as compared to HG. To do so, we followed a single nationwide cohort of more than 5,000 patients across 13 regions. Our data showed that consistent with intuition, referring true positives out of the system decreases the prevalence of STDR in the cohort over time. This decrease happens because the number of true positives was detected with high sensitivity, and their removal presumably leaves behind more difficult examples (false negatives). As the cohort continued to develop STDR, new-onset STDR (i.e., more subtle cases) developed, further enriching the cohort with diagnostically challenging STDR cases. This enrichment for difficult cases may help explain the decreased sensitivity and positive predictive value of both DL and HG in the second screening.

The degree to which this enrichment happens is dependent on the sensitivity of the screening modality. For example, HG had a lower sensitivity in the first screening, which led to a larger number of false negative cases (185 vs. 35) that entered the second screening, and correspondingly a relative 33% higher STDR prevalence at the second screening (HG: 6.8% vs. DL: 5.1%). Thus, we expect that more accurate DL methods or experienced HG will lead to fewer false negatives but a more rapid increase in case difficulty at follow-up visits.

False negative cases are also concerning because they represent cases missed for treatment referral and are thus at risk of vision loss. While such misses are inevitable, this proportion was relatively small when expressed as a fraction of the entire screening population: 0.5-0.6% for DL and about 3% for HG. In addition, most false negative cases were DME, with generally less than 10% being severe NPDR or PDR in both DL and HG cohorts. The increase in proportion of severe NPDR or PDR in false negatives in the second screening might reflect the limitation of both modalities in being able to detect subtle changes of new severe NPDR or PDR compared to DME. Because “screen-negative” cases (i.e., true negatives and false negatives) comprise more than 85% of the cohort, having retina specialists overread all such cases is likely impractical. To help improve the ability to detect more difficult or subtle STDR cases, better DL algorithms or continuing education, monitoring, and audits of HG may be useful. Nonetheless, the particularly low incidence of false negatives by DL (and even then with DME representing the majority) suggests DL-based biennial DR screening can be clinically acceptable.

In contrast to false negatives, decreasing the rate of false positives might improve costs. In our setup, overreads were performed for every “screen-positive” (i.e., true positives and false positives). Reducing the rate of this “over-triggering” can reduce the need for such overreads and help scale DR screening. We anticipate that our detailed data can aid future cost-effectiveness or cost-utility analyses into evaluating DL for DR screening and cost-benefit analysis of overreads vs. unnecessary referrals.

Our study contains some limitations. First, as a retrospective study, our inclusion criteria and desire to study longitudinal outcomes required patients to have retinal photographs in two screenings. Such a cohort may not fully reflect real-world screening settings. Similarly, cohorts do not remain static, but instead, newly diagnosed patients with diabetes enter the screening program on an on-going basis. Though we have not accounted for this, the proportion of new patients with diabetes is expected to be small (estimated at 5% by the National Health Security Office in Thailand). Second, though we expect the trends observed in increasing diagnostic-difficulty and decreasing sensitivity to hold over subsequent screenings (beyond the second), we have not conducted that analysis in this study. Third, the performance of HG may be underestimated because they did not have images from previous screenings available, whereas access to previous images is common practice in real-world settings. Finally, patients with moderate NPDR without DME were included in our biennial screening cohort. Although this group accounted for only 5% of the patients in the first screening, 25% of them progressed to STDR in the second screening. It may be advisable to stratify DR screening patients by their expected risk of developing STDR [27, 30, 31] and initiating biennial screening only for patients in the low-risk group.

The DL used in our study was developed to categorize DR severity and detect DME, and hence, the evaluation of the algorithm's capability to detect other retinal diseases was not possible. The development of DL models that are capable of detecting multiple retinal conditions is an important area of active research. Similarly, the ungradable images in our simulated cohort were “referred” based on our program's standard protocol, with the reason being that many contain cataracts. In this regard, future development of an AI that can more accurately detect DR in the eyes with cataracts may be valuable to reduce the overall referral burden.

## 5. Conclusion

In a longitudinal follow-up of a biennial DR screening cohort, DL performed well, with higher sensitivities and positive predictive values than HG in both the first and second screening. This was despite a case spectrum shift as STDR cases were referred for treatment, and the remaining false negative cases were joined by new STDR cases, both of which were presumably more subtle and difficult to detect. To reduce unnecessary referrals, further studies on health economics could provide guidance on whether expert overreading is required for all “screen-positive” cases.

## Figures and Tables

**Table 1:** Demographic characteristics of patients with diabetes in the first and second screening, including the prevalence of each diabetic retinopathy severity level and diabetic macular edema.

Characteristics	First screening, DL and HG (*n* = 5,738)	Second screening, DL (*n* = 4,148)	Second screening, HG (*n* = 4,263)
Age, years, mean ± SD	57.27 ± 10.44	56.51 ± 10.52	56.53 ± 10.51
Female, *n* (%)	3,945 (68.8%)	2,874 (69.3%)	2,951 (69.2%)
Hypertension, *n* (%)	3,895 (67.9%)	2,855 (68.8%)	2,921 (68.5%)
FBS, mg/dL in mean ± SD	151.26 ± 52.83	150.48 ± 50.97	150.65 ± 51.23
No NPDR, *n* (%)	4,152 (72.36%)	3,239 (78.09%)	3,256 (76.38%)
Mild NPDR no DME, *n* (%)	589 (10.26%)	448 (10.80%)	449 (10.53%)
Moderate NPDR no DME, *n* (%)	293 (5.11%)	250 (6.03%)	269 (6.31%)
Severe NPDR no DME, *n* (%)	6 (0.10%)	7 (0.17%)	6 (0.14%)
PDR no DME, *n* (%)	47 (0.82%)	11 (0.27%)	17 (0.40%)
DME, *n* (%)	651 (11.35%)	193 (4.65%)	266 (6.24%)

DL: deep learning; HG: trained human graders; FBS: fasting blood sugar; NPDR: nonproliferative diabetic retinopathy; PDR: proliferative diabetic retinopathy; DME: diabetic macular edema. The prevalence of each DR severity level and DME in each cohort was determined by the reference standard.

**Table 2:** The number of patients with sight-threatening diabetic retinopathy including the screening outcomes in the first and second screening determined by each modality.

Modality	Metric	First screening	Second screening	Difference (%)	*p* value
DL	No. of patients	5,738	4,148	*n*/*a*	
	No. STDR (%)	704 (12.27%)	211 (5.09%)	-7.18	<0.001^∗^
	No. graded as STDR (%)	771 (13.44%)	274 (6.61%)	-6.83	<0.001^∗^
	Sensitivity (95% CI)	95.03 (93.42-96.63)	90.05 (86.01-94.09)	-4.98	0.008^∗^
	Specificity (95% CI)	97.97 (97.58-98.36)	97.87 (97.42-98.32)	-0.10	0.742
	PPV (95% CI)	86.77 (84.38-89.16)	69.34 (63.88-74.8)	-17.43	<0.001^∗^
	NPV (95% CI)	99.3 (99.06-99.53)	99.46 (99.23-99.69)	+0.16	0.318
	Accuracy (95% CI)	97.61 (97.22-98.01)	97.47 (96.99-97.95)	-0.14	0.657
HG	No. of patients	5,738	4,263	*n*/*a*	
	No. STDR (%)	704 (12.27%)	289 (6.78%)	-5.49	<0.001^∗^
	No. graded as STDR (%)	590 (10.28%)	224 (5.25%)	-5.03	<0.001^∗^
	Sensitivity (95% CI)	73.72 (70.47-76.97)	57.09 (51.39-62.8)	-16.63	<0.001^∗^
	Specificity (95% CI)	98.59 (98.26-98.92)	98.52 (98.14-98.89)	-0.07	0.753
	PPV (95% CI)	87.97 (85.34-90.59)	73.66 (67.89-79.43)	-14.31	<0.001^∗^
	NPV (95% CI)	96.41 (95.9-96.91)	96.93 (96.4-97.46)	+0.52	0.169
	Accuracy (95% CI)	95.54 (95-96.07)	95.71 (95.1-96.32)	+0.17	0.681

STDR: sight-threatening diabetic retinopathy; PPV: positive predictive value; NPV: negative predictive value; DL: deep learning; HG: trained human graders; CI: confidence interval. *p* value was calculated from chi-squared test for the difference between the first and second screening. ^∗^*p* value < 0.05.

**Table 3:** The number of patients in the first and second screening in each cell of the contingency table (true positive, false positive, true negative, and false negative) for detecting sight threatening diabetic retinopathy by each modality.

Modality	Metric	First screening	Second screening	Difference (%)	*p* value
DL	No. of patients	5,738	4,148	*n*/*a*	
	True positives	669 (11.66%)	190 (4.58%)	-7.08%	<0.001^∗^
	False positives	102 (1.78%)	84 (2.03%)	+0.25%	0.3671
	True negatives	4,932 (85.95%)	3,853 (92.89%)	+6.94%	0.001^∗^
	False negatives	35 (0.61%)	21 (0.51%)	-0.1%	0.5139
HG	No. of patients	5,738	4,263	*n*/a	
	True positives	519 (9.04%)	165 (3.87%)	-5.17%	0.001^∗^
	False positives	71 (1.24%)	59 (1.38%)	+0.14%	0.5410
	True negatives	4,963 (86.49%)	3,915 (91.84%)	+5.35%	<0.001^∗^
	False negatives	185 (3.22%)	124 (2.91%)	-0.31%	0.3755

STDR: sight-threatening diabetic retinopathy; DL: deep learning; HG: human graders; *p* value was calculated from chi-squared test for the difference between the first and second screening. ^∗^*p* value < 0.05.

**Table 4:** The 2-year progression of patients from baseline retinopathy severity levels in the first screening into sight-threatening diabetic retinopathy detected by each modality in the second screening.

Baseline retinopathy levels at the first screening, *n*	Number of patients with STDR in the second screening, per reference standard (%)	DL, number of patients with STDR in the second screening, per reference standard (%)	HG, number of patients with STDR in the second screening, per reference standard (%)
No retinopathy, 4,136	71 (1.72%)	128 (3.09%)	73 (1.76%)
Mild NPDR, 584	50 (8.56%)	57 (9.76%)	41 (7.02%)
Moderate NPDR, 293	74 (25.26%)	97 (33.11%)	57 (19.45%)
Total of all non-STDR severity levels, 5,013	195 (3.89%)	282 (5.63%)	171 (3.41%)

STDR: sight-threatening diabetic retinopathy; NPDR: nonproliferative diabetic retinopathy; DL: deep learning; HG: trained human graders. *p* < 0.001 for the proportions of STDR of patients in the different baseline severity levels from the first screening in each modality.

## Data Availability

The deidentified data underlying this study may be available from DR screening programs of Rajavithi Hospital, Lamphun Hospital, Somdejphrajaotaksin Maharaj Hospital, Sawanpracharak Hospital, Nakhon Nayok Hospital, Photharam Hospital, Prapokklao Hospital, Mahasarakham Hospital, Nongbualamphu Hospital, Pakchong-nana Hospital, Mukdahan Hospital, Suratthani Hospital, Sungaikolok Hospital, and Bangkok Metropolitan Administration Public Health Center 7, but restrictions apply. Researchers interested in collaborating should contact the corresponding author.

## Additional Points

*Code Availability*. Machine learning models were developed in prior work and deployed using standard software libraries and scripts in TensorFlow. Custom deployment code was specific to our computing infrastructure and mainly used for data processing.

## Supplementary Materials

Supplementary Materials The supplementary information includes tables showing the number of patients with STDR or false negatives classified into severe NPDR/PDR and DME; the number of patients with non-STDR or false positive classified into no/mild NPDR and moderate NPDR without DME; the number of the eyes with STDR, including DME and severe NPDR/PDR, and screening outcomes in the first and second screening determined by each modality.
