# Supplementary material for: Longitudinal Screening for Diabetic Retinopathy in a Nationwide Screening Program: Comparing Deep Learning and Human Graders
Source: J Diabetes Res. 2020 Dec 15;2020:8839376. doi: 10.1155/2020/8839376 (PMC7758133; doi:10.1155/2020/8839376)
Supplement: Supplementary Materials — The supplementary information includes tables showing the number of patients with STDR or false negatives classified into severe NPDR/PDR and DME; the number of patients with non-STDR or false positive classified into no/mild NPDR and moderate NPDR without DME; the number of the eyes with STDR, including DME and severe NPDR/PDR, and screening outcomes in the first and second screening determined by each modality. [file 8839376.f1.docx]

## Supplementary Information

**Supplementary Table 1. The number of patients with sight-threatening diabetic retinopathy or false negatives classified into severe non-proliferative diabetic retinopathy/proliferative diabetic retinopathy and diabetic macular edema.**

|  |  | Modality | Total | Severe NPDR/PDR | DME |
| --- | --- | --- | --- | --- | --- |
| All STDR | First screening | Both cohorts | 704 | 53 (7.53%) | 651 (92.47%) |
|  | Second screening | DL | 211 | 18 (8.53%) | 193 (91.47%) |
|  |  | HG | 289 | 23 (7.96%) | 266 (92.04%) |
| FN | First screening | DL | 35 | 2 (5.71%) | 33 (94.29%) |
|  |  | HG | 185 | 13 (7.03%) | 172 (92.97%) |
|  | Second screening | DL | 11 | 4 (36.36%) | 7 (63.64%) |
|  |  | HG | 124 | 14 (11.29%) | 110 (88.71%) |

STDR = sight-threatening diabetic retinopathy; NPDR = non-proliferative diabetic retinopathy; PDR = proliferative diabetic retinopathy; DME = diabetic macular edema; DL = deep learning cohort; HG = trained human graders cohort; FN = false negatives.

**Supplementary Table 2. The number of patients with non-sight-threatening diabetic retinopathy or false positive classified into no/mild non-proliferative diabetic retinopathy and moderate non-proliferative diabetic retinopathy without diabetic macular edema.**

|  |  | Modality | Total | No/Mild NPDR | Moderate NPDR without DME |
| --- | --- | --- | --- | --- | --- |
| All non-STDR | First screening | Both cohorts | 5,034 | 4,741 (94.18%) | 293 (5.82%) |
|  | Second screening | DL | 3,937 | 3,687 (93.65%) | 250 (6.35%) |
|  |  | HG | 3,974 | 3,705 (93.23%) | 269 (6.77%) |
| FP | First screening | DL | 102 | 36 (35.29%) | 66 (64.71%) |
|  |  | HG | 71 | 58 (81.69%) | 13 (18.31%) |
|  | Second screening | DL | 84 | 39 (46.43%) | 45 (53.57%) |
|  |  | HG | 59 | 47 (79.66%) | 12 (20.34%) |

STDR = sight-threatening diabetic retinopathy; NPDR = non-proliferative diabetic retinopathy; DL = deep learning cohort; HG = human graders cohort; FP = false positives.

**Supplementary Table 3. The number of eyes with sight-threatening diabetic retinopathy, including diabetic macular edema and severe non-proliferative/proliferative diabetic retinopathy, and screening outcomes in the first and second screening determined by each modality**

|  | Modality | Metric | First screening | Second screening |
| --- | --- | --- | --- | --- |
| STDR    Eyes/total 1,001/12,412 (8.06%) | DL | Eyes/total | 1,150/12,412 (0.09%) | 475/9,905 (0.05%) |
|  |  | Sensitivity (95%CI) | 94.81 (93.43-96.18) | 88.98 (85.76-92.2) |
|  |  | Specificity (95%CI) | 98.24 (98.00-98.48) | 98.41 (98.16-98.66) |
|  |  | PPV (95%CI) | 82.52 (80.33-84.72) | 68.00 (63.80-72.2) |
|  |  | NPV (95%CI) | 99.54 (99.41-99.66) | 99.58 (99.44-99.71) |
|  |  | Accuracy (95%CI) | 97.96 (97.71-98.21) | 98.06 (97.79-98.33) |
|  | HG | Eyes/total | 747/12,412 (0.06%) | 366/10,148 (0.04%) |
|  |  | Sensitivity (95%CI) | 65.33 (62.39-68.28) | 53.59 (49.28-57.90) |
|  |  | Specificity (95%CI) | 99.18 (99.02-99.35) | 99.07 (98.87-99.26) |
|  |  | PPV (95%CI) | 87.55 (85.18-89.92) | 75.41 (71.00-79.82) |
|  |  | NPV (95%CI) | 97.03 (96.72-97.33) | 97.56 (97.25-97.86) |
|  |  | Accuracy (95%CI) | 96.46 (96.13-96.78) | 96.76 (96.41-97.10) |
| DME    Eyes/total 901/12,417 (7.26%) | DL | Eyes/total | 1,062/12,417 (0.09%) | 474/9,983 (0.05%) |
|  |  | Sensitivity (95%CI) | 94.78 (93.33-96.24) | 90.75 (87.70-93.80) |
|  |  | Specificity (95%CI) | 98.19 (97.95-98.44) | 98.34 (98.08-98.59) |
|  |  | PPV (95%CI) | 80.41 (78.03-82.80) | 66.24 (61.99-70.50) |
|  |  | NPV (95%CI) | 99.59 (99.47-99.70) | 99.66 (99.55-99.78) |
|  |  | Accuracy (95%CI) | 97.95 (97.7-98.20) | 98.08 (97.81-98.35) |
|  | HG | Eyes/total | 648/12,417 (0.05%) | 342/10,223 (0.03%) |
|  |  | Sensitivity (95%CI) | 62.71 (59.55-65.87) | 53.86 (49.46-58.27) |
|  |  | Specificity (95%CI) | 99.28 (99.12-99.43) | 99.21 (99.03-99.38) |
|  |  | PPV (95%CI) | 87.19 (84.62-89.76) | 77.49 (73.06-81.91) |
|  |  | NPV (95%CI) | 97.15 (96.84-97.45) | 97.70 (97.41-98.00) |
|  |  | Accuracy (95%CI) | 96.63 (96.31-96.94) | 97.03 (96.70-97.36) |
| Severe NPDR or PDR    Eyes/total 285/12,882 (2.21%) | DL | Eyes/total | 563/12,882 (0.04%) | 350/11,224 (0.03%) |
|  |  | Sensitivity (95%CI) | 94.74 (92.14-97.33) | 86.99 (81.05-92.94) |
|  |  | Specificity (95%CI) | 97.67 (97.41-97.94) | 97.81 (97.54-98.08) |
|  |  | PPV (95%CI) | 47.96 (43.83-52.08) | 30.57 (25.74-35.40) |
|  |  | NPV (95%CI) | 99.88 (99.82-99.94) | 99.85 (99.78-99.92) |
|  |  | Accuracy (95%CI) | 97.61 (97.35-97.87) | 97.69 (97.41-97.97) |
|  | HG | Eyes/total | 202/12,882 (0.02%) | 111/11,306 (0.01%) |
|  |  | Sensitivity (95%CI) | 60.35 (54.67-66.03) | 45.06 (37.40-52.72) |
|  |  | Specificity (95%CI) | 99.76 (99.68-99.85) | 99.66 (99.55-99.77) |
|  |  | PPV (95%CI) | 85.15 (80.24-90.05) | 65.77 (56.94-74.59) |
|  |  | NPV (95%CI) | 99.11 (98.95-99.27) | 99.21 (99.04-99.37) |
|  |  | Accuracy (95%CI) | 98.89 (98.71-99.07) | 98.88 (98.68-99.07) |

STDR = sight-threatening diabetic retinopathy; PPV = positive predictive value; NPV = negative predictive value; DL= deep learning; HG = human graders.
